# High SPOCK1 Expression Is Associated with Advanced Stage, T Value, and Gleason Grade in Prostate Cancer

**DOI:** 10.3390/medicina55070343

**Published:** 2019-07-05

**Authors:** Mei-Ling Chen, Cheng-Ju Ho, Chung-Min Yeh, Sung-Lang Chen, Wen-Wei Sung, Shao-Chuan Wang, Chih-Jung Chen

**Affiliations:** 1 Department of Surgical Pathology, Changhua Christian Hospital, Changhua 50006, Taiwan; 2 Department of Urology, Chung Shan Medical University Hospital, Taichung 40201, Taiwan; 3 Department of Medical Technology, Jen-Teh Junior College of Medicine, Nursing and Management, Miaoli 35664, Taiwan; 4 School of Medicine, Chung Shan Medical University, Taichung 40201, Taiwan; 5 Institute of Medicine, Chung Shan Medical University, Taichung 40201, Taiwan

**Keywords:** SPOCK1, prostate cancer, Gleason grade, survival

## Abstract

*Background and objectives*: Prostate cancer (PCa) is a common malignancy in males and has a relatively slower progression than other cancers. Our goal was to evaluate the clinical role of SPARC (secreted protein acidic and cysteine rich, osteonectin), cwcv, and kazal-like domains’ proteoglycan 1 (SPOCK1) in PCa. *Materials and Methods*: SPOCK1 expression was studied through the immunohistochemical staining of specimens from 71 patients with PCa. The correlation between SPOCK1 expression and clinicopathological features was quantitatively analyzed. We used Kaplan–Meier analysis and Cox proportional hazard models to analyze the prognostic value. *Results*: Of 71 PCa patients, high SPOCK1 expression was more likely to be seen in those with an advanced stage (*p* = 0.018) of the disease and an advanced tumor (T) value (*p* = 0.014). Patients in Gleason grade groups 3 and 4 had significantly higher SPOCK1 expression (*p* = 0.044 and 0.003, respectively) compared to those of Gleason grade group 1. However, this trend was not observed in patients in Gleason grade group 5. For the survival analysis, although it was not statistically significant, patients with a high SPOCK1 expression had a shorter median overall survival (6.2 years) compared to those with low expression (7.8 years). *Conclusions*: High SPOCK1 expression may be related to advanced clinicopathological features and possibly a poor prognosis. Further analysis with a larger patient base would help clarify this issue.

## 1. Introduction

Prostate cancer (PCa)—a common malignancy and a major cause of cancer deaths in men—has a slower progression than other cancers [1,2]. Cancers of the prostate, lung and bronchus, and colorectum account for approximately 50% of all newly diagnosed cancers in men; notably, PCa alone accounts for 28% (238,590 patients) [3]. The five-year relative survival rate among patients with cancer that is either confined to the prostate (localized) or only has regional spread is 100%; by contrast, it is 29.3% among those diagnosed with distant metastases. Thus, even with the slower progression of PCa, an effort toward the early detection of metastasis is needed to identify a better prognosticator and enable better oncology-targeted therapies.

The trend toward personalized treatment is universal; however, it is still in a fledgling state for the field of PCa [4]. In PCa, secreted protein acidic and cysteine rich, osteonectin (SPARC), cwcv, and kazal-like domains’ proteoglycan 1 (SPOCK1) increases with the progression of human PCa through epithelial-to-mesenchymal transition signaling, which contributes to proliferative and metastatic activities in vitro and in vivo [5]. Overexpression of SPOCK1 was observed in both naïve PCa and castration-resistant prostate cancer tissues [6]. SPOCK1 encodes a calcium-binding matricellular glycoprotein that belongs to the SPARC family [7]. SPOCK1 is known to play crucial roles in regulating proliferation, cell cycle progression, apoptosis, adhesion, cell matrix interaction, and metastasis [5,8]. Previous studies have revealed that SPOCK1 has importance in the progression of pilocytic astrocytoma [9] and hepatocellular carcinoma [10]. In addition to the brain and liver, the higher expression of SPOCK1 in urothelial carcinoma correlates with a higher pathological tumor value, lymph node metastasis, higher histological grade, and more vascular and perineurial invasion [11]. The role of SPOCK1 in PCa shows that it acts as a critical mediator in tumor growth and metastasis [5,12].

The aim of our study was to demonstrate and clarify the correlation between SPOCK1 expression and overall survival, prognosis, and clinicopathological factors in PCa. Our results may provide a novel aspect of SPOCK1 to the clinical parameters of PCa.

## 2. Methods

### 2.1. Patients

Our study examined 71 tumor samples from patients with PCa. The cancers were staged according to the American Joint Committee on Cancer (AJCC) Cancer Staging Manual. The clinicopathological features that were assessed in this study, including histology, tumor node metastasis (TNM) stage, and Gleason score, were confirmed by two pathologists. Patients with primary PCa and tissue available from a bio-bank were included in this study. Those with either missing data or tissue loss during the staining procedure were excluded from this study. The study was approved by the Institutional Review Board and the Ethics Committee of the Changhua Christian Hospital, Changhua, Taiwan (CCH IRB 161117, approved on 18 January 2017).

### 2.2. Immunohistochemical Staining of SPOCK1

Immunohistochemical staining was performed at the Department of Surgical Pathology, Changhua Christian Hospital, as described previously [13,14], using an anti-human SPOCK1 antibody (SPOCK1 antibody, 1:1100 dilution; Abcam, ab83768). The sections were placed on coated slides, de-paraffinized with xylene, and rehydrated through serial dilutions of alcohol, followed by washings with phosphate buffered saline (pH = 7.2). Endogenous peroxidase activity was blocked with 3% H_2_O_2_. Antigen retrieval was performed via boiling in citrate buffer (10 mM) for 20 min. The sections were incubated with an anti-human antibody for 20 min at room temperature and then thoroughly washed. The immunoreaction was visualized using a polymer-based MACH4 DAB (3,3’-diaminobenzidine) Detection Kit (Biocare Medical, Concord, CA, USA), according to the manufacturer’s instructions, to obtain optimal immunoreactivity and the fewest background artifacts. The slides were incubated with a horseradish peroxidase/Fab polymer conjugate for another 30 min. The sites of peroxidase activity were visualized using 3,31-diamino-benzidine tetrahydrochloride (Biocare Medical, CA, USA) as the substrate for 5 min, after which they were counterstained with hematoxylin (Biocare Medical, Concord, CA, USA). Phosphate buffered saline was used instead of primary antibodies as a negative control. Immunoreactivity scores were analyzed by three pathologists using a previously described scoring protocol, which was defined as a cell staining intensity (0–3) that was multiplied by the percentage of stained cells (0–100%), leading to scores from 0–300 [15]. The pathologists were blind to the prognostic data. A final agreement was obtained for each score by having all three evaluators view the specimens.

### 2.3. Patient and Public Involvement

This study analyzed cancer tissues from a de-linked database. Therefore, we neither informed nor disseminated information to the patients about either the research question, outcome measures, or results. The patients were not involved in the study’s design, recruitment, or conduct. There was no patient adviser for the contributorship statement.

### 2.4. Statistical Analysis

The χ^2^ test and independent sample t-test were applied for the data analysis. The associations between SPOCK1 expression and patient survival were estimated using univariate analysis, the Kaplan–Meier method, and the log-rank test [14,15]. Potential confounders, including age, Gleason grade group, and stage, were adjusted using Cox regression models. All statistical analyses were conducted using SPSS statistical software (version 15.0; SPSS, Inc., Chicago, IL). All statistical tests were two-sided, and values of *p* < 0.05 were considered statistically significant.

## 3. Results

**Figure 1:**
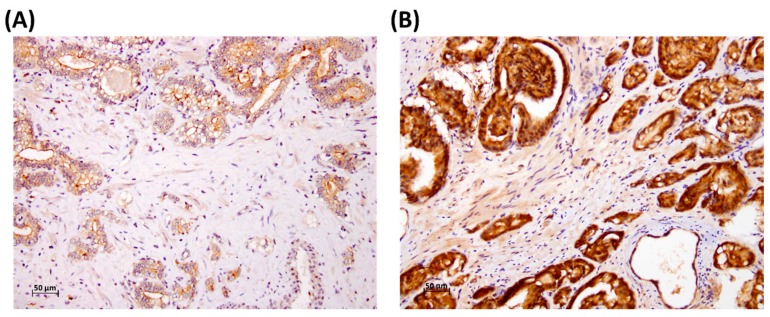
Representative immunostaining of SPARC (secreted protein acidic and cysteine rich osteonectin), cwcv, and kazal-like domains’ proteoglycan 1 (SPOCK1) of prostate cancer (PCa) specimens with (**A**) low and (**B**) high expression.

A total 71 patients who had undergone a radical prostatectomy were analyzed. The representative staining figures are shown in Figure 1. The relationship between SPOCK1 expression and age revealed no statistical significance (73.2 ± 5.8 versus 75.5 ± 7.4, *p* = 0.151, Table 1). The Gleason grade groups, which were divided into two groups of 1 + 2 and 3 + 4 + 5, also showed no difference in SPOCK1 expression (*p* = 0.370). In addition, there was no difference in SPOCK1 expression whether there was either nearby lymph node metastasis or distal metastasis (*p* = 0.489 and 0.315, respectively). Statistically significant differences for SPOCK1 expression were noted in the pathological disease stage and the tumor (T) value (*p* = 0.018 and 0.014, respectively).

The SPOCK1 expression score was higher in advanced cancer stages compared with earlier cancer stages (254 ± 60, *p* = 0.020, Table 2). While it was not the case in Gleason grade groups 2 and 5, the SPOCK1 expression scores were significantly higher in groups 3 and 4 (*p* = 0.044 and 0.003, respectively) than they were in group 1, using group 1 as a reference.

In the univariate analysis, the Gleason grade groups (3 + 4 + 5 and 1 + 2) had a meaningful influence on the overall survival of PCa patients (*p* = 0.001, Table 3), with a median survival of 4.1 years and 9.6 years, respectively (hazard ratio (HR): 3.700, 95% confidence interval (CI) 1.769–7.737). A similar phenomenon was observed in advanced tumor value (III + IV and I + II), with a median survival of 4.9 years and 9.4 years, respectively (HR: 2.419, 95% CI 1.075–5.444). By contrast, there seemed to be no influence of either age or SPOCK1 expression level on overall survival. When adjusting for age, Gleason grade group, and stage in the multivariate analysis (Table 4), a worse effect was noted on overall survival in the more advanced Gleason grade group compared with the earlier group (HR: 3.322, 95% CI 1.531–7.209, *p* = 0.002). Pathological stage showed no influence on overall survival after the adjusted analysis (*p* = 0.136), and there was also no obvious influence from either age or SPOCK1 expression (*p* = 0.651 and 0.405, respectively).

**Figure 2:**
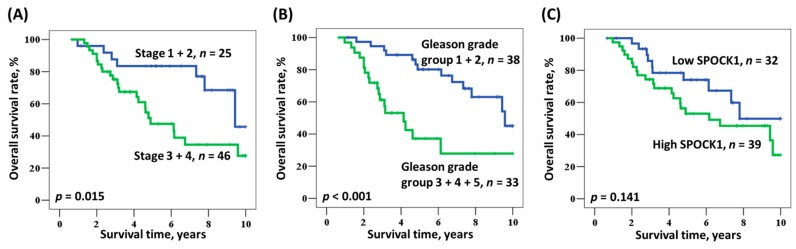
Kaplan–Meier actuarial analysis of overall survival according to (**A**) stage, (**B**) Gleason grade group, and (**C**) SPOCK1 expression.

Figure 2A demonstrates the Kaplan–Meier analysis of overall survival according to stage. The pathological stage 1 + 2 group had a better overall survival trend compared with the stage 3 + 4 group (*p* = 0.015). The lesser Gleason grade group (1 + 2) also showed a similar trend (*p* < 0.001), which is demonstrated in Figure 2B. Lower overall survival was noted in the high SPOCK1 expression group compared with the low expression group, but it was not statistically significant (*p* = 0.141, Figure 2C).

## 4. Discussion

A significant difference in overall survival has been noted between the local and distal forms of PCa. The early detection of metastasis in a distant lymph node, bone, or other organ becomes crucial not only for improved clinical outcomes, but also regarding treatment differences that are based on staging. In this study, we investigated SPOCK1 expression along with patient age, Gleason grade, stage, T value, N value, and M value. It is well known that an elevated expression of SPOCK1 is accompanied by a tendency toward an advanced T value and lymphatic invasion in urothelial carcinoma [11]. We found a similar pattern in PCa. With a more advanced tumor value, the ratio of high SPOCK1 expression became more dominant, which supports the result of an advanced cancer stage accompanying high SPOCK1 expression (Table 1). A previous study suggested that SPOCK1 may play a role as a promoter of PCa metastasis [6]. Even with no statistical significance for a correlation between SPOCK1 expression and the N value and M value, a larger number of cases with high SPOCK1 expression were observed in those with lymph node metastasis and distal metastasis. However, more cases are needed to support the prior findings.

We further quantified the SPOCK1 expression level with a score from 0–300 and analyzed the parameters of stage and Gleason grade group, which are shown in Table 2. As per our expectation, an advanced cancer stage was accompanied by a higher SPOCK1 score, and it showed an escalation with elevated Gleason grade. However, the SPOCK1 expression score for Gleason grade 5 was the lowest for all groups. Therefore, we can hypothesize that another carcinogenesis pathway takes control in poorly differentiated PCa rather than the SPOCK1-related pathway as multiple signal pathways have been associated with PCa carcinogenesis [16]. Further investigation is needed to elucidate and understand the sophisticated interactions between carcinogenesis pathways and their roles in cancer progression and metastasis.

Comparisons between the Gleason 1 + 2 and the Gleason 3 + 4 + 5 groups regarding the overall survival of PCa showed a 5.5-year difference (9.6 years versus 4.1 years, respectively, Table 3 and Table 4). Nevertheless, a more advanced cancer stage did not affect overall survival in the multivariate analysis after adjusting for age, Gleason grade group, and stage (95% CI, 0.811–4.679, *p* = 0.136, Table 4). This result corresponds with the characteristically slow progression of PCa and intimates the importance of histologic differentiation in prognosis. Our study results demonstrated the overexpression of SPOCK1 in advanced T value and Gleason grade groups compared with earlier stages; this was based on a previous study of these factors when compared to benign tissue [17]. Although the SPOCK1 expression level seemed to have a less direct influence on overall survival in the variate analysis, the tendency toward a worse prognosis in high expression is shown in Figure 1. The aforementioned facts may suggest that SPOCK1 expression in PCa has the potential to be a prognostic factor and an indicator of tumor malignancy [5].

The limitation of our study is the small study patient group. Because of treatment strategy selectivity and the patient group being composed mainly of the elderly, the case cumulative velocity was slower than it would be with other malignancies. Otherwise, there is no investigation of the molecular mechanism of SPOCK1 and its target genes, as well as a therapeutic intervention. In conclusion, this study presented the relationships between SPOCK1 expression and several clinicopathological parameters in PCa patients. SPOCK1 may play a role as an independent prognostic factor for PCa, but further investigation is needed to support this hypothesis.

## 5. Conclusions

High SPOCK1 expression may be related to both advanced stage and advanced T value in PCa. Patients with a high SPOCK1 expression had shorter median overall survival compared to those with low expression, although it was not statistically significant. Further analysis with a larger number of patients would help clarify this issue.

## Figures and Tables

**Table 1:** Relationships between SPOCK1 expression and clinical parameters in PCa patients.

		SPOCK1 Expression		
Parameters	Case Number	Low	High	*p* Value
Age (years)		73.2 ± 5.8	75.5 ± 7.4	0.151
Gleason grade group				
1 + 2	38	19 (50.0)	19 (50.0)	0.370
3 + 4 + 5	33	13 (39.4)	20 (60.6)	
Stage				
I + II	25	16 (64.0)	9 (36.0)	0.018
III + IV	46	16 (34.8)	30 (65.2)	
Tumor (T) value				
1	13	10 (76.9)	3 (23.1)	0.014 *
2 + 3 + 4	58	22 (37.9)	36 (62.1)	
Node (N) value				
0	55	26 (47.3)	29 (52.7)	0.489
1 + 2	16	6 (37.5)	10 (62.5)	
Metastasis (M) value				
0	42	21 (50.0)	21 (50.0)	0.315
1	29	11 (37.9)	18 (62.1)	

* Fisher’s exact test (two-sided).

**Table 2:** SPOCK1 expression score according to stage and Gleason grade group in PCa patients.

Parameters	SPOCK1 Expression*	*p* Value
Stage		
1+2	216 ± 73	Reference
3+4	254 ± 60	0.020
Gleason grade group		
1	229 ± 65	Reference
2	248 ± 65	0.375
3	270 ± 46	0.044
4	285 ± 17	0.003
5	210 ± 82	0.454

* Mean ± standard deviation (SD).

**Table 3:** Univariate analysis of the influence of various parameters on the overall survival of PCa patients.

		Overall Survival			
Parameter	Category	Medium Survival (Years)	Hazard Ratio (HR)	95% Confidence Interval (CI)	*p* Value
Age, years	≥74/<74	7.8/11.3	1.335	0.651–2.738	0.430
Gleason grade group	3 + 4 + 5/1 + 2	4.1/9.6	3.700	1.769–7.737	0.001
Stage	III + IV/I + II	4.9/9.4	2.419	1.075–5.444	0.033
SPOCK1	High/Low	6.2/7.8	1.750	0.823–3.720	0.146

**Table 4:** Multivariate analysis of the influence of various parameters on the overall survival of PCa patients.

		Overall Survival			
Parameter	Category	Mean Survival (Years)	HR	95% CI	*p* Value
Age, years	≥74/<74	7.8/11.3	1.193	0.555–2.562	0.651
Gleason grade group	3 + 4 + 5/1 + 2	4.1/9.6	3.322	1.531–7.209	0.002
Stage	III + IV/I + II	4.9/9.4	1.948	0.811–4.679 *	0.136 *
SPOCK1	High/Low	6.2/7.8	1.411	0.627–3.174	0.405

* Adjusted for age, Gleason grade group, and stage.

## Abbreviations

CI: confidence interval; HR: hazard ratio; mCRPC: metastatic, castration-resistant prostate cancer; PARP: poly ADP-ribose polymerase; PCa: prostate cancer; SPOCK1: SPARC (osteonectin), cwcv and kazal like domains proteoglycan 1.
